# Allo-HSCT compared with immunosuppressive therapy for acquired aplastic anemia: a system review and meta-analysis

**DOI:** 10.1186/s12865-020-0340-x

**Published:** 2020-03-06

**Authors:** Yangmin Zhu, Qingyan Gao, Jing Hu, Xu Liu, Dongrui Guan, Fengkui Zhang

**Affiliations:** Department of Therapeutic Center of Anemia, Institute of Hematology and Blood Diseases Hospital, Chinese Academy of Medical Science & Peking Union Medical College (CAMS & PUMC), Tianjin, China

**Keywords:** Aplastic anemia and bone marrow failure, Transplantation, Red cell disorders

## Abstract

**Background:**

Allogeneic hematopoietic stem cell transplantation (allo-HSCT) and immunosuppressive therapy (IST) are two major competing treatment strategies for acquired aplastic anemia (AA). Whether allo-HSCT is superior to IST as a front-line treatment for patients with AA has been a subject of debate. To compare the efficacy and safety of allo-HSCT with that of IST as a front-line treatment for patients with AA, we performed a meta-analysis of available studies that examined the impact of the two major competing treatment strategies for AA.

**Results:**

Fifteen studies including a total of 5336 patients were included in the meta-analysis. The pooled hazard ratio (HR) for overall survival (OS) was 0.4 (95% CI 0.074–0.733, *P* = 0.016, I^2^ = 58.8%) and the pooled HR for failure-free survival (FFS) was 1.962 (95% CI 1.43–2.493, *P* = 0.000, I^2^ = 0%). The pooled relative risk (RR) for overall response rate (ORR) was 1.691 (95% CI 1.433–1.996, *P* = 0.000, I^2^ = 11.6%).

**Conclusion:**

Although survival was significantly longer among AA patients undergoing first-line allo-HSCT compared to those undergoing first-line IST, the selection of initial treatment for patients with newly diagnosed AA still requires comprehensive evaluation of donor availability, patient age, expected quality of life, risk of disease relapse or clonal evolution after IST, and potential use of adjunctive eltrombopag.

## Background

Acquired aplastic anemia (AA) is a rare hematologic disease characterized by a profound deficit of hematopoietic stem and progenitor cells, bone marrow hypocellularity, and peripheral blood pancytopenia. It mainly affects children, young adults, and those over 60 years of age. The estimated incidence rate of AA ranges from 0.7 to 4.1 per million population each year, and it appears to be two to three times higher in Asia than in Europe and North America [1, 2]. Although the occurrence of AA can be partly explained by some drugs, chemicals, viruses, and other external factors, the majority of cases are idiopathic [3]. The underlying pathophysiology is thought to be an aberrant autoimmune reaction involving the T-cell-mediated destruction of hematopoietic cells [4]. Major symptoms are infections, hemorrhage, and symptoms of anemia. Symptoms may be severe and life-threatening or minor enough to not require transfusion support. The survival rate for AA has markedly improved in the past four decades because of advances in hematopoietic stem cell transplantation (HSCT), immunosuppressive drugs, and supportive care.

According to the 2015 Guidelines for the Diagnosis and Management of AA (GDMAA) of the British Committee for Standards in Haematology (BCSH), first-line immunosuppressive therapy (IST) is a combination of antithymocyte globulin (ATG) and ciclosporin (CsA), indicated for non-severe AA (NSAA) patients who are suffer with transfusion dependency, encountering infections, recurrent bleeding, or hope for improved quality of life; severe AA (SAA) or very severe AA (VSAA) patients in the absence of an HLA-matched sibling donor (MSD); or SAA/VSAA patients > 35–50 years of age [5]. Although IST is effective at alleviating pancytopenia in a number of patients, it is not effective in all cases. In addition, it has been recognized that a part of patients treated with IST develop clonal hematopoiesis or somatic mutations that lead to myelodysplastic syndrome (MDS) or acute myeloid leukemia (AML) [6]. Furthermore, the high-risk recurrence of AA makes this treatment strategy a second choice behind HSCT from an HLA-matched family donor.

The 2015 GDMAA recommends first-line allogeneic hematopoietic stem cell transplantation (allo-HSCT) from the bone marrow of an MSD for treating SAA in young and adult patients who have an MSD. Unrelated donor (URD) HSCT is indicated for SAA after failure to respond to at least one course of nontransplant IST. Alternative donor allo-HSCT using either cord blood (CB) or a haploidentical family donor (HID) may be recommended for patients after failure to respond to IST and in the absence of a MSD or a suitably matched URD [5]. Although matched related donor (MRD) HSCT can be successfully conducted after the failure of IST or after evolution to MDS/AML, overall survival is decreased when transplantation is used as second-line treatment [7]. Moreover, the outcomes after allo-HSCT from an URD have steadily improved over the past three decades. Recent data have revealed similar outcomes for upfront-unrelated and matched sibling HSCT for pediatric AA, which supports the recommendation for first-line treatment with an URD-HSCT for children who lack an MSD [8, 9]. In the past, alternative donor HSCT was another salvage choice for cure in patients with refractory AA after IST, but morbidity and mortality from graft failure and complications of graft-versus-host disease (GVHD) have limited clinical applications for this approach. With the improvements in transplantation technology and management of GVHD, haploidentical-HSCT (HID-HSCT) has become a viable alternative treatment for patients who lack an MRD. The successful application of posttransplant cyclophosphamide (PT-CY) for HLA-haploidentical grafts for AA patients generated high rates of engraftment, low rates of transplant-related mortality, low rates of GVHD, and eradication of pre-existing clonal diseases [10]. The evidence of improved long-term survival after HID-HSCT supports the potential role of HID-HSCT as a first-line therapy.

To compare the efficacy and safety of allo-HSCT with that of IST as a front-line treatment for patients with AA, we performed a meta-analysis of available studies that examined the impact of the two major competing treatment strategies for AA.

## Results

### Included studies

**Fig. 1:**
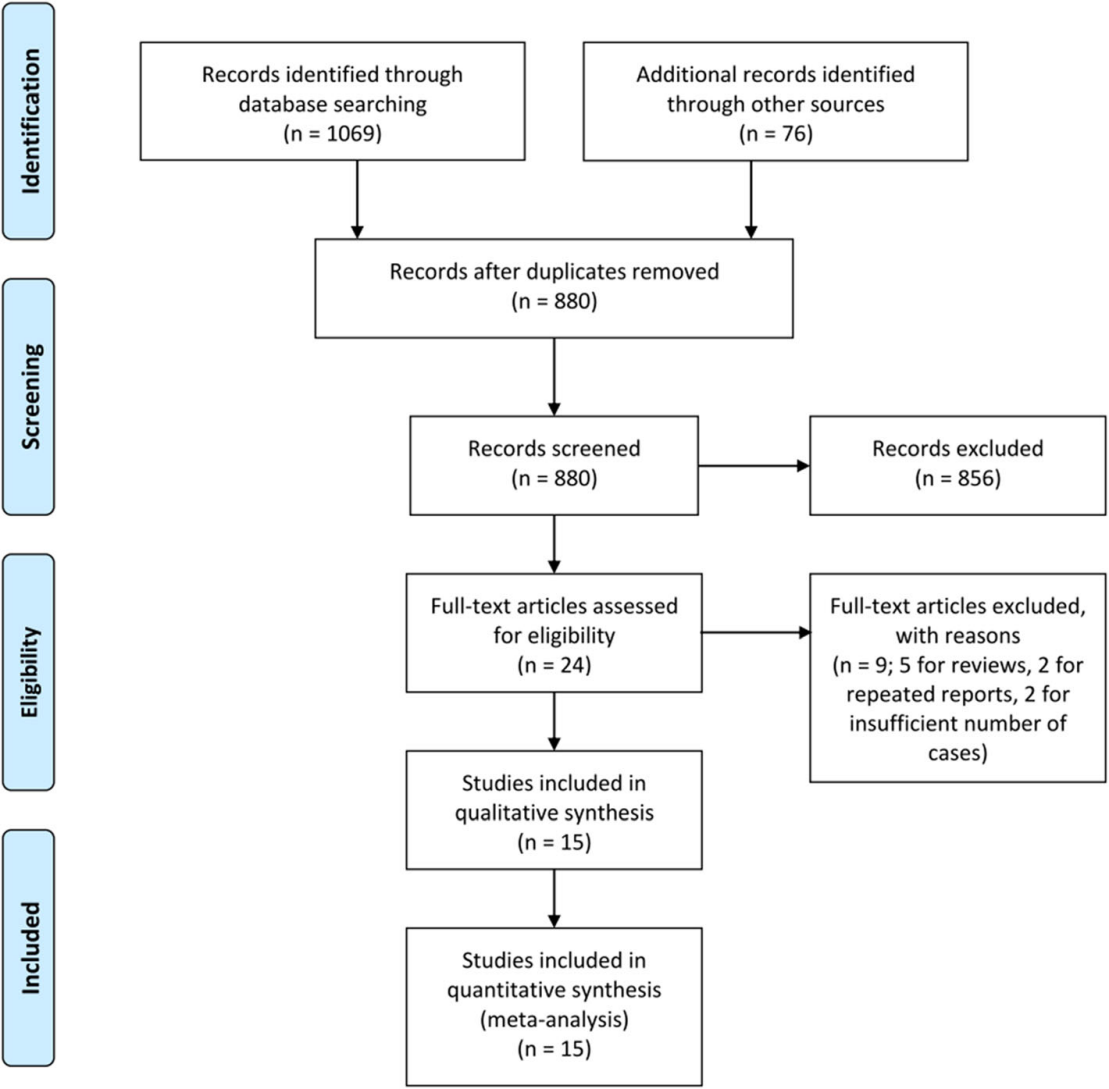
Study selection flow diagram

We gained 880 citations from the electronic database and manual screen and 27 potentially related citations were retrieved as full-text or were checked for more detailed investigation (Fig. 1). Five reviews and three abstracts were excluded, two studies were excluded for insufficient patient number, and two were excluded for potentially repeated reports. Ultimately, 15 studies with 5336 patients met the predefined selection criteria (Table 1).

**Table 1:** Characteristics and quality assessment of individual studies included in the meta-analysis

First author (Publication year)	Duration of study	Population	Disease severity	Median age (HSCT/IST)	Number of patients (HSCT/IST)	Donor	Stem cell source	Conditioning program	Prevention of GVHD	IST drugs	Follow-up time (HSCT/IST)	Selection/Comparability/Exposure	NOS Score
Kojima et al. (2000) [11]	1984–1998	Children	SAA + VSAA + NSAA	10 (0–16), 9 (1–17)	37/63	MRD	BM	Cy + TLI, Cy + ATG ± TLI, Cy + TLI + TBI	MTX + CsA, CsA	ATG/ALG + HD-Steroids, ATG + CsA	89 (6–166), 82 (6–186); (Month)	****/**/**	8
Fouladi et al. (2000) [12]	1987–1997	Children	SAA	8.8 (2.1–15.9), 9.8 (1.3–16.6)	21/20	MRD	BM	Cy + TBI and others	MTX + CsA, CsA, CsA + Pred	ATG ± CsA	4.3 (3.3–6.7), 3 (0.2–6.7); (Year)	***/**/**	7
Choi et al. (2017) [13]	1998–2012	Children	SAA	9.3 (0.6–17.2), 8.5 (1.3–14.1)	23/19	MUD	BM/PBSCs/CB	Flu + Cy ± TBI, Bu + Cy + ATG, Cy + TBI, Cy + ATG	MTX + CsA or Tac, CsA, others	ATG + CsA	NR	***/**/**	7
Cheng et al. (2017)	2007–2016	Children	SAA	8 (2–17), 6 (4–16)	28/24	HID	BM + PBSCs	Bu + Cy + ATG	CSA + MTX + MMF	ATG + CsA	37.9 (8.7–108.3), 54.8 (8.9–115.7); (Month)	***/**/**	7
Yoshida et al. (2014) [14]	1992–2009	Children	SAA	11 (0–16), 9 (0–16)	213/386	MRD	BM	Cy ± Irradiation, CY + ATG ± Irradiation, Flu + Cy ± Irradiation, Flu + Cy + ATG ± Irradiation and others	MTX + CsA or Tac	ATG + CsA ± G-CSF	101 (18–213), 106 (22–224); (Month)	***/**/**	7
Dufour et al. (2015) [15]	2000–2009	Children	SAA	NR	396/167	MRD	BM/PBSCs	Cy, Cy + Flu ± ATG and others	CsA + MTX	ATG + CsA	NR	***/**/**	7
Yang et al. (2019) [16]	2012–2016	Children	SAA	13 (4–18), 12 (4–17)	20/29	HID	BM/PBSCs, BM + PBSCs	Cy + ATG ± Flu, Flu + Cy + Bu + ATG	CsA + MTX + MMF	ATG + CsA	NR	***/**/**	7
Xu et al. (2018)	2009–2017	Adult	SAA	28 (18–49), 32 (18–62)	28/32	HID	BM + PBSCs	Bu + Cy + ATG	MTX + CsA + MMF	ATG + CsA	24.7 (6.1–103), 20.2 (3.2–96.0); (For alive) (Month)	***/**/**	7
Ahn et al. (2003) [17]	1990–2001	Adult	SAA	28 (14–43), NR	64/156	MRD	BM	Cy + ATG, Cy + TLI	MTX + CsA, CsA	ATG + CsA, ATG + HD-Steroids, CsA	NR	***/**/**	7
Kim et al. (2003) [18]	1990–1999	Adult	SAA	22 (14–43), 34 (15–75)	22/74	MRD	BM	Cy + TBI, Cy + ATG, Bu + Cy	MTX + CsA	ATG/ALG, ATG + CsA	NR	***/**/**	7
Ellis et al. (2002) [19]	1977–1999	Adult + Children	SAA	22 (6–59), 55 (9–78)	15/16	MRD	BM	Cy ± Irradiation	MTX, MTX + CsA	ATG, ATG + CsA	4.3 (0.2–242.2), 52.5 (1.1–165.4); (Month)	***/**/**	7
Ghavamzadeh et al. (2004) [20]	1990–2001	Adult + Children	SAA	19, 25 (Mean)	29/24	MRD	BM/PBSCs	CY ± ALG	CsA	CsA ± ALG	878 (24–2750), 403 (NR); (Mean) (Day)	***/**/**	7
Viollier et al. (2005) [21]	1976–1999	Adult + Children	SAA + VSAA + NSAA	19 (2–55), 23 (2–74)	52/155	MRD	BM/PBSCs	CY ± ATG	MTX, MTX + CsA	ATG, ATG + CsA	11.5 (2–22), 11.3 (0.2–22); (Year)	****/**/**	8
Locasciulli et al. (2007) [22]	1991–2002	Adult + Children	SAA	18.7 (1–67), 23.5 (1–94)	1567/912	MRD (85%) + MMRD (3%) + MUD (10%)	BM	Cy, Cy + ALG, Cy + Irradiation (TBI, TLI, TAI)	CsA ± MTX, some not specified	ALG + CsA ± G-CSF	41 (24–155), 54.4 (14–144); (For alive) (Month)	***/**/**	7
George et al. (2015) [23]	1985–2013	Adult + Children	SAA + VSAA + NSAA	22 (3–57), 30.1 (1.5–74)	214/530	MRD + MMRD (Percentage NR)	BM/PBSCs	Flu + Cy, Cy ± ATG, Flu + Bu and others	MTX + CsA, PT-CY	ATG/ALG + CsA	36 (6–197), 38 (1–84); (Month)	****/**/**	8

*MMRD* Mismatched related donor, *Cy* Cyclophosphamide, *Bu* Busulfan, *TLI* Total lymphoid irradiation, *TBI* Total body irradiation, *TAI* Thoraco-abdominal irradiation, *CsA* Cyclosporin A, *G-CSF* Granulocyte colony-stimulating factor, *Pred* Prednisone, *Tac* Tacrolimus, *MMF* Mycophenolate mofetil, *HD-Steroids* High-dose steroids, *NR* Not reported. Selection: representativeness of exposed cohort, selection of no exposed cohort, ascertainment of exposure, outcome not present at start; comparability: comparability of cohorts on the basis of the design or analyses; outcome: assessment of outcome, follow-up length, follow-up adequacy. Newcastle-Ottawa Quality Assessment Scale: a study was awarded a maximum of one star (*) for meeting each criterion, a maximum of two stars (**) was given for comparability

### Characteristics of the included studies

All studies reported the outcomes of AA patients treated with first-line allo-HSCT or IST [11–25]. The allo-HSCT group was divided into two subgroups: BMT-MRD and BMT-MUD. In one study, the BMT-matched unrelated donor (MUD) subgroup was deleted for having an insufficient patient number (*n* = 5) [12]. The non-transplant group consisted of two subgroups, cyclosporin alone and androgen. In one study, the androgen subgroup was deleted for receiving non-IST treatment [20]. Those studies were published between 2000 and 2019, and all were retrospective studies. The case collection period ranged from 1976 to 2016. Sample sizes ranged from 31 to 2479 (15 to 1567 in the allo-HSCT group and 16 to 912 in the IST group). The study population was children in 7 studies, adults in 3 studies, and both children and adults in 5 studies. Most studies included only SAA patients, but 3 studies included SAA, VSAA, and NSAA patients. The median age was 8–28 years in the allo-HSCT group and 6–55 years in the IST group. Only one study reported mean age, and one study only reported the median age of the allo-HSCT group. One study reported neither the median age nor the sex ratio. For the allo-HSCT group, the donor was an MRD in 9 studies, an HID in 3 studies, and an MUD in 1 study. One study used mainly MRD donors together with mismatched related donor (MMRD) or MUD. Most of the studies adopted a cyclophosphamide-based regimen as a conditioning program. Prophylaxis against GVHD mainly consisted of CsA and methotrexate (MTX). Stem cell sources consisted of bone marrow (BM), peripheral blood stem cells (PBSCs), and a few patients with CB. The IST drugs mainly consisted of ATG or ALG combined with CsA. The quality of the studies in the analyses was high, with a mean overall NOS assessment score of 7.2 (range, 7–8).

### Primary outcomes

**Fig. 2:**
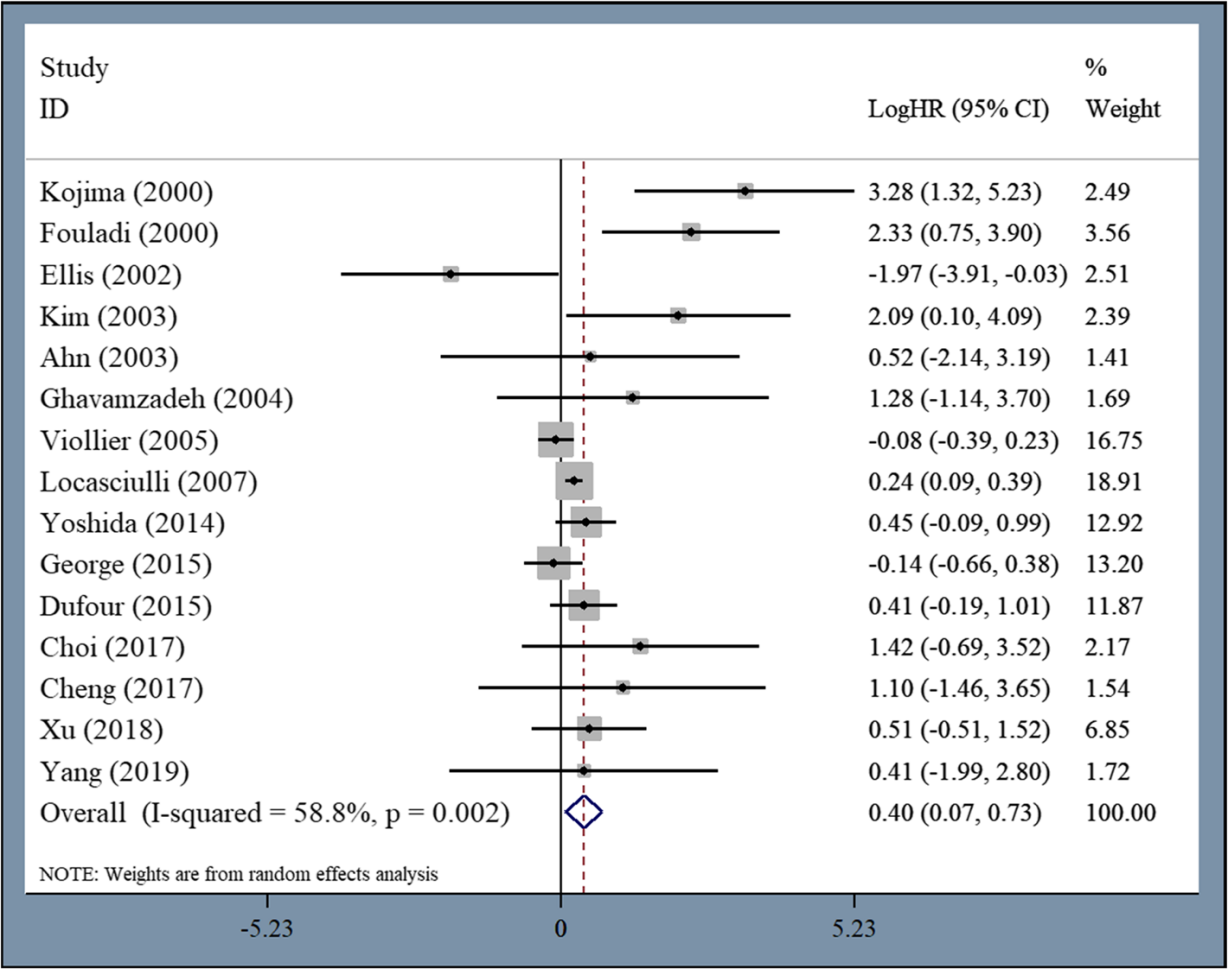
Significantly longer OS among patients undergoing first-line allo-HSCT compared to first-line IST

All studies reported OS. The pooled HR for OS was 0.4 (95% CI 0.074–0.733, *P* = 0.016, I^2^ = 58.8%) (Fig. 2). These data indicate that first-line allo-HSCT is significantly superior to IST for patients with AA. However, we found marked heterogeneity in the pooled HR for OS, and a subsequent sensitivity analysis revealed four studies had caused significant heterogeneity. After excluding these four studies [19, 21–23], the pooled HR for OS from the remaining 11 studies with 1875 patients was 0.955 (95% CI 0.443–1.468, *P* = 0.000, I^2^ = 34.7%) (Additional file 1: Fig. S1), which still indicates the superiority of first-line allo-HSCT over IST for patients with AA. When OS was analyzed according to the publication year, there was a trend towards longer survival among patients undergoing first-line allo-HSCT compared to IST between 2010 and 2019 (HR = 0.286, 95% CI -0.008–0.58, *P* = 0.057, I^2^ = 0%). Data analysis prior to 2010 was not performed due to the extreme heterogeneity of the results. When OS was analyzed according to the study population, there was a trend towards longer survival among adult patients undergoing first-line allo-HSCT compared to those undergoing IST (HR = 0.801, 95% CI -0.056–1.658, *P* = 0.067, I^2^ = 0%). Although large heterogeneity was detected, first-line allo-HSCT was significantly superior to first-line IST for children with AA (HR = 1.068, 95% CI 0.358–1.779, *P* = 0.003, I^2^ = 54%). We then analyzed OS according to the disease severity, and there was significantly longer survival among SAA patients undergoing first-line allo-HSCT compared to first-line IST (HR = 0.506, 95% CI 0.13–0.881, *P* = 0.008, I^2^ = 38.8%). We further analyzed OS according to the donor type and found no difference in survival between patients undergoing first-line haploidentical-HSCT and IST (HR = 0.563, 95% CI -0.315–1.441, *P* = 0.209, I^2^ = 0%). However, significantly longer survival with large heterogeneity was observed in patients undergoing first-line MRD-HSCT compared to IST (HR = 0.711, 95% CI 0.053–1.37, *P* = 0.034, I^2^ = 73.4%).

**Fig. 3:**
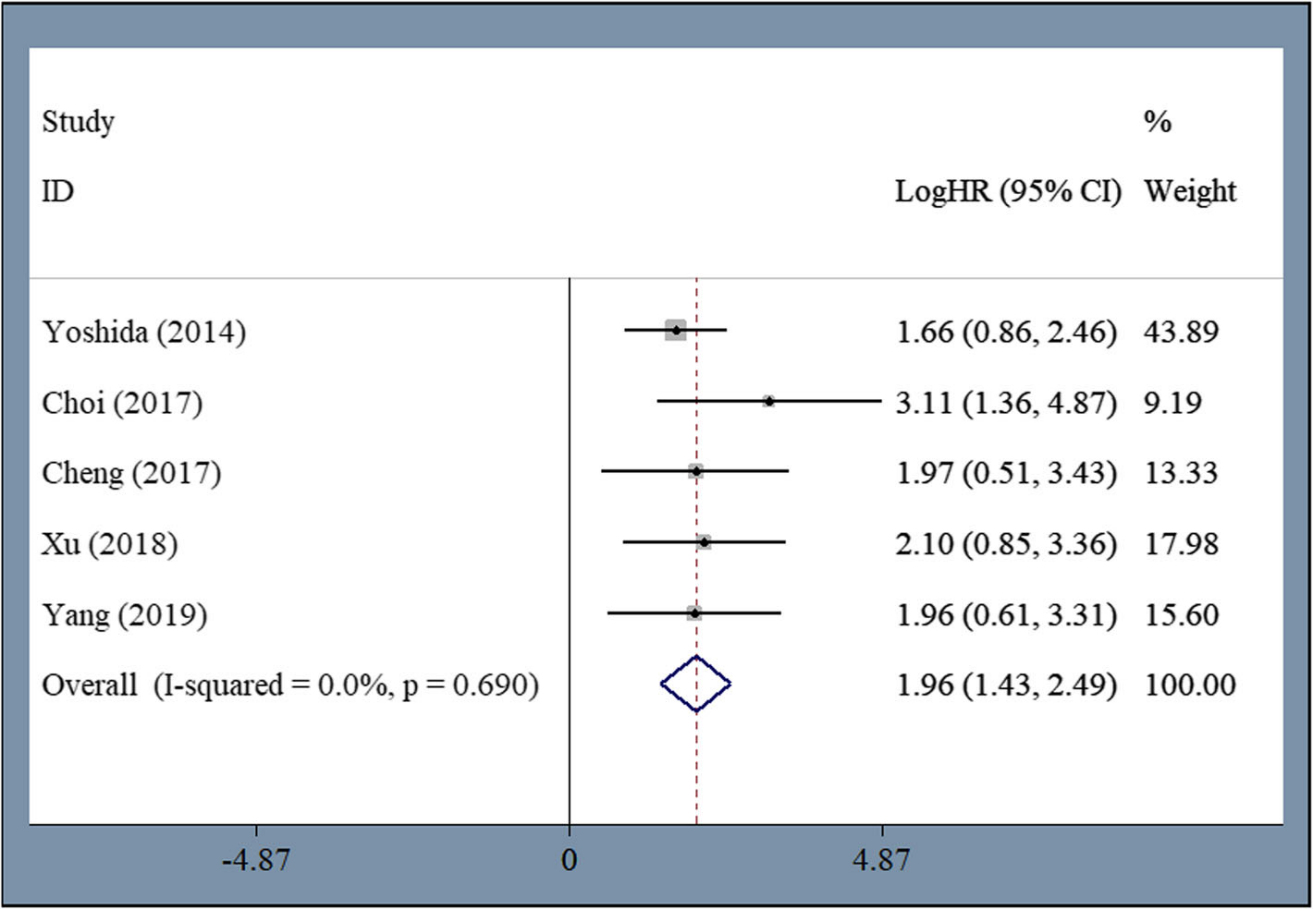
Significantly longer FFS among patients undergoing first-line allo-HSCT compared to first-line IST

Five studies including 802 patients reported FFS in the meta-analysis. The pooled HR for FFS was 1.962 (95% CI 1.43–2.493, *P* = 0.000, I^2^ = 0%) (Fig. 3), which indicates that first-line allo-HSCT was significantly superior to IST for patients with AA in regard to FFS.

### Secondary outcomes

Three studies including 314 patients reported comparable ORR in the meta-analysis. The pooled RR for ORR was 1.691 (95% CI 1.433–1.996, *P* = 0.000, I^2^ = 11.6%). These data indicate that first-line allo-HSCT caused a higher treatment response compared to first-line IST for patients with AA. Four studies reported comparable CRs but they were not included in the data analysis due to their extreme heterogeneity. Only two studies including 238 patients reported comparable TRM. There was a significantly higher TRM among patients undergoing first-line allo-HSCT compared to IST (pooled RR 3.98, 95% CI 1.911–8.29, *P* = 0.000, I^2^ = 0%).

**Fig. 4:**
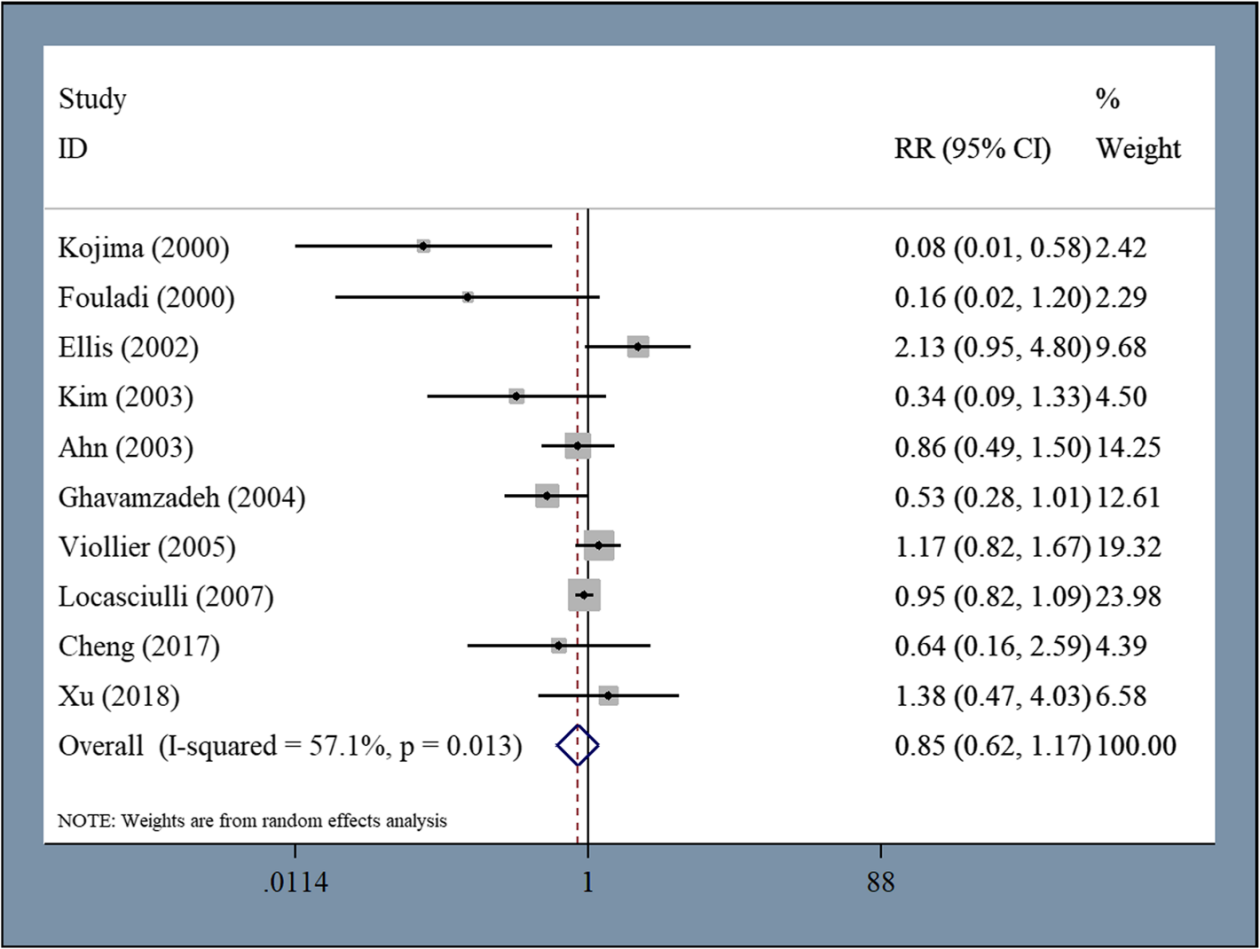
No difference was observed in all-cause mortality between patients undergoing first-line allo-HSCT and those undergoing first-line IST

Ten studies including 3339 patients reported all-cause mortality. For these studies, the pooled RR was 0.851 (95% CI 0.618–1.174, *P* = 0.327, I^2^ = 57.1%) (Fig. 4). No difference in all-cause mortality was observed between patients who received first-line allo-HSCT and those who received IST. Subsequent analysis revealed no difference in mortality resulting from hemorrhage (pooled RR = 0.491, 95% CI 0.199–1.208, *P* = 0.122, I^2^ = 37%) but there was significantly higher mortality resulting from infection among patients who received first-line IST compared to first-line allo-HSCT (pooled RR = 1.378, 95% CI 1.081–1.757, *P* = 0.01, I^2^ = 0%). The median rate of engraftment was 96% (range 80–100%) and the median rate of graft failure was 5% (range 1–13%) for patients who received first-line allo-HSCT. Acute GVHD developed in 42.5% (range 23–100%) of patients, 25% (range 4–48%) of which were grade II–IV. Chronic GVHD developed in 30% (range 7–61%) of patients, 28% (range 6–39%) of patients had limited cGVHD, 6.5% (range 3–30%) of patients had extensive cGVHD, and 7% (range 2–27%) died of GVHD. Of the patients that received first-line IST, 4% (range 1–19%) developed MDS/AML and 15% (range 9.5–45%) experienced relapse. Only two studies reported the incidence of paroxysmal nocturnal hemoglobinuria (PNH) after first-line allo-HSCT or IST (6 and 12%, respectively).

## Disscussion

Acquired aplastic anemia (AA) is a serious hematologic disorder characterized by peripheral blood pancytopenia caused by bone marrow failure. The pathogenesis of this disease is thought to be the destruction of hematopoietic stem cells by autoimmunity. Allo-HSCT and IST using a combination of ATG and CsA have been the cornerstone of therapy for both SAA and NSAA patients since the 1970s. MRD-HSCT is now recommended as a first-line treatment for young and adult patients who have an MRD. MUD-HSCT is indicated for SAA patients after failure to respond to IST. First-line IST is a therapeutic option for patients in the absence of an MRD or with old age. However, this treatment approach is based on the results of comparative studies conducted mainly in the 1980s. Transplantation success for AA patients with an MRD has improved considerably over the past three decades, with a 75 to 80% chance of long-term cure. Now there is controversy concerning the upper age limit for MRD-HSCT as a first-line treatment because results vary in different series. Data from the European Group for Blood and Marrow Transplantation (EBMT) database revealed similar outcomes for patients in the age ranges of 20 to 30 years, 30 to 40 years, and 40 to 50 years [26]. Although treating a patient with transplantation alone in the case of IST failure is an appealing strategy, outcomes in patients undergoing transplantation after failing IST are worse than those in patients treated with first-line MRD-HSCT [7]. During the last two decades, the outcome of allo-HSCT for AA patients with an MUD has also improved significantly, suggesting that this treatment should be given an increased role in the treatment of children and young adults with AA who are without an MRD. Recent studies have revealed similar long-term OS after transplantation from an MRD compared to an MUD [8]. MUD-HSCT is therefore recommended as the first-line treatment for AA patients eligible for transplant but lacking an MRD. Furthermore, it may even be appropriate for some older adults to proceed with first-line MUD-HSCT. Alternative donor transplantation with PT-CY for AA has shown satisfactory outcomes, with high rates of engraftment, low rates of transplant-related mortality, low rates of GVHD, and eradication of pre-existing clonal diseases [10]. This program allowed expansion of the donor pool to allow use of HIDs and MRDs, and it is also under development as a first-line therapy in appropriate patient circumstances. Altogether, the evidence for improved long-term survival after allo-HSCT from various donor types supports the broader role of allo-HSCT as a first-line therapy. To comprehensively evaluate the efficacy and safety of allo-HSCT compared with IST as a front-line treatment for patients with AA, we performed a meta-analysis that examined the impact of the two major competing treatment strategies.

Our meta-analysis demonstrated significantly longer OS and FFS, as well as a higher response rate, for patients who underwent first-line allo-HSCT compared to first-line IST. However, this outcome should be interpreted with caution. All studies were non-randomized retrospective studies, and there was significant selection bias in the analysis. Young patients with severe disease and with available donors were more likely to receive transplantation, whereas older patients, patients with no available donor, or patients with severe complications tended to receive IST. Moreover, non-transplant centers would probably apply more likely IST while transplant centers would tend to apply allo-HSCT, which would add further bias. Most of the studies reported the outcomes of patients given first-line MRD-HSCT compared to those of patients given IST. Only two and three studies reported the outcomes of first-line MUD-HSCT and HID-HSCT compared to IST, respectively. Evidence for improved long-term survival with first-line allo-HSCT over IST is more robust for patients given first-line MRD-HSCT. Moreover, we found no difference in survival between patients undergoing first-line HID-HSCT and first-line IST in our subgroup analysis. This is consistent with the present controversial treatment situation. In China, HID-HSCT has been advocated as a first-line treatment for children with AA [24], but in the United States and Europe, HID-HSCT is regarded as an experimental treatment for the relatively limited number of cases reported with unknown long-term effects of complicated regimens and a mismatched immune system. In addition, we observed a great disparity in FFS for patients treated with allo-HSCT versus IST. Treatment failure was defined as death, no response, disease progression, or relapse in patients who received IST. However, the time of response evaluation was at 6 months after completion of treatment in most studies. In fact, the number of patients that achieved response increased over time for patients who received IST. Some patients acquired a defined response as late as 24 months after the completion of treatment [19]. In this situation, FFS may have been underestimated for patients who received IST. In addition, although overall survival was reduced when allo-HSCT was used as a second-line treatment compared to a first-line treatment in some studies, the survival advantage of allo-HSCT as a first-line treatment over allo-HSCT as a second-line treatment after the failure of IST or IST as a first-line treatment is still unknown.

Eltrombopag is a synthetic small molecule mimetic of thrombopoietin used for patients who remain pancytopenic after treatment with IST. Recently, it was also under evaluation for use in combination with IST for the treatment of SAA as a first-line therapy. In a recent study, the addition of eltrombopag to IST was associated with markedly higher rates of hematologic response among patients with SAA than in a historical cohort. The overall response rate at 6 months was 94% in one of three cohorts; at a median follow-up of 2 years, the survival rate was 97% [27]. However, to date, there has been no study comparing the efficacy of first-line allo-HSCT and first-line IST in combination with eltrombopag. In our opinion, this nontransplant combination therapy strategy may counterbalance the survival advantage of allo-HSCT in indicated patients with AA.

Some researchers have recommended revision of the guidelines for the first-line treatment of patients with newly diagnosed SAA. Allo-HSCT should be recommended as an initial treatment for newly diagnosed SAA patients. HLA typing should be conducted to identify a marrow donor among family members or in the donor registries at the time of diagnosis. The priority of donor source for allo-HSCT is MRD, MUD, follow by HID if a MUD is not rapidly accessible. Each of these donors may be superior to IST because of the long-term high-risk for disease recurrence and secondary MDS/AML with the use of IST for patients with AA. On the contrary, allo-HSCT is associated with high cure rate, a low risk for disease recurrence or the development of clonal disorders, and a relatively low risk for GVHD [28]. It has been well recognized that a high incidence of somatic mutations that lead to the development of clonal evolution and MDS/AML can be detected after IST treatment. However, it should be noted that clonal hematopoiesis on the situation of bone marrow failure does not reliably predict the development of clinical diagnosed diseases. In one study, only a small minority of AA patients with DNMT3A or ASXL1 mutations developed MDS during the follow-up time [29]. This is consistent with what was found during our meta-analysis. We found that a median of 15% (9.5–45%) of patients in our included studies experienced relapse, which is lower than the 37–38% of patients reported in other studies [30, 31]. This discrepancy may be due to different patient populations, the definition of response, treatment protocols, and the limited follow-up time in some studies. It has been suggested that IST should not be discontinued after response to therapy in patients with NSAA and SAA due to the high risk of relapse. Indefinite administration of low dose CsA may reduce the incidence of relapse [32]. For patients who received allo-HSCT, we found a higher incidence of GVHD, but the incidence of grade II–IV aGVHD, extensive cGVHD, and death caused by GVHD was relatively low.

Given that we saw no difference in all-cause mortality between patients who received allo-HSCT and IST, the quality of life assessment is a matter of cardinal significance for comparing these two treatment strategies. The late adverse events of allo-HSCT are of major consideration for patients who survive long-term after transplantation, but they are often left out of the comparison of the two treatment strategies. For example, fertility has been shown to be reduced when alkylating agents are combined with total-body irradiation during conditioning. Children born from patients after allo-HSCT are at increased risk for developing genetic diseases or congenital anomalies [33]. There is a methodology termed the quality-adjusted time without symptoms and toxicity (Q-TWiST) which allows for evaluate quality of life by retrospective analysis the time a patient spent in different health states, supposing that quality of life is reliance on different health state [34]. This assessment procedure can provide a detailed view on the result of the two treatment modalities by integrating quality of life parameters into the comparison, for instance, transfusion requirement, drug demand, adverse events, GVHD or clonal evolution. Studies used Q-TWiST revealed that patients treated with allo-HSCT take more time cured from AA, whereas IST patients have more transfusion requirements, medication demand, close medical care and spend more time in cost-intensive periods, in spite of similar overall survival and event-free survival were observed [21].

## Conclusions

Although survival is significantly longer among AA patients undergoing first-line allo-HSCT compared to first-line IST, the selection of initial treatment for patients with newly diagnosed AA requires a comprehensive evaluation of donor availability, age, expected quality of life, risk of disease relapse or clonal evolution after IST, and the use of adjunctive eltrombopag. Our meta-analysis highlights the need for prospective studies to examine the role of these two treatment modalities.

## Methods

### Search strategy and study selection

We searched the Cochrane Registry of Controlled Trials databases, Embase, and Medline from January 2000 to March 2019. We also searched the reference lists of all identified studies as well as related articles including review papers. We used the following search terms: (Allogeneic hematopoietic stem cell transplantation OR Marrow transplantation) AND (Immunosuppressive therapy OR antithymocyte OR antilymphocyte globulin) AND (aplastic anemia). Studies comparing allo-HSCT with IST as a first-line therapy for patients with AA were included. Two reviewers (JH and QYG) respectively screened the titles and abstracts of all identified studies to evaluate their eligibility for inclusion. Only studies with full-text and a sample size > 30 patients were included.

### Data extraction and quality assessment

Two reviewers (YMZ and XL) respectively extracted the data from each study including publication year, study region, first author, period of enrollment, patient number, median age, conditioning program, hematopoietic stem cell source, prevention of GVHD, follow-up time, and study outcome. Disagreements between the two reviewers were solved via discussion. Two researchers (QYG and DRG) evaluated the quality of the included studies using the Newcastle-Ottawa Scale (NOS) [35]. The NOS consists of eight items classified into three dimensions including selection (four items), comparability (one item), and exposure (three items). A study can be awarded a maximum of one star for each item within the selection and exposure categories and a maximum of two stars can be given for comparability. The quality of the studies was classified into high quality (scores 7–9), intermediate quality (scores 4–6), and low quality (scores 1–3) studies.

### Definition of outcomes

Primary outcomes of this study were overall survival (OS) and failure-free survival (FFS). Secondary outcomes were overall response rate (ORR), complete response rate (CR), treatment-related mortality (TRM), rates of engraftment, graft failure and GVHD, incidence of MDS/AML after IST, and cause of death. OS was defined as the time to death from any cause or at the last follow-up (censored). FFS was defined as survival with response or censored. No response, disease progression, relapse or death were defined as treatment failures in patients who received IST. Primary or secondary graft failure, relapse or death were defined as treatment failure in patients who received transplantation. In the IST cohort, evaluation of response was performed at 6 months following IST. Complete response (CR) was defined as an absolute neutrophil count of more than 1.5 × 10^9^/L, a platelet count of more than 100 × 10^9^/L and a hemoglobin level of more than 110 g/L. Partial response (PR) was defined as an absolute neutrophil count of more than 0.5 × 10^9^/L, a platelet count more than 20 × 10^9^/L, a hemoglobin level of more than 80 g/L, and no requirement of blood transfusion.

### Statistical analyses

All statistical analyses were performed using Stata software (ver. 14.0, StataCorp, College Station, TX, USA). Descriptive statistics were presented as median and range for non-comparative data. We measured the hazard ratios (HRs) for OS and FFS and relative risk (RR) for other outcomes. The HR and their 95% confidence intervals (CIs) were estimated by the method created by Tierney et al. [36]. The statistical heterogeneity of the studies was evaluated using the chi-square-based Q-test and quantified with the I^2^ statistic: (1) no heterogeneity for I^2^ = 0–25%, (2) moderate heterogeneity for I^2^ = 25–50%, (3) large heterogeneity for I^2^ = 50–75%, (4) extreme heterogeneity for I^2^ = 75–100%). A fixed-effect model with the inverse variance approach was used to calculate estimates of the pooled HR or RR and their respective 95% CIs. In the situation of moderate or large heterogeneity (I^2^ = 25–75% or *P*-value < 0.1), a random-effects model using the DerSimonian and Laird method was utilized.

## Supplementary information

**Additional file 1: Fig. S1.** Significantly longer OS among patients undergoing first-line allo-HSCT compared to first-line IST after excluding four studies with high heterogeneity.

## Data Availability

The datasets used and/or analysed during the current study available from the corresponding author on reasonable request.

## Abbreviations

AA: Aplastic anemia
allo-HSCT: Allogeneic hematopoietic stem cell transplantation
GVHD: Graft-versus-host disease
HID: Haploidentical family donor
IST: Immunosuppressive therapy
MSD: HLA-matched sibling
URD: Unrelated donor
