# Supplementary material for: Allo-HSCT compared with immunosuppressive therapy for acquired aplastic anemia: a system review and meta-analysis
Source: BMC Immunol. 2020 Mar 6;21:10. doi: 10.1186/s12865-020-0340-x (PMC7059290; doi:10.1186/s12865-020-0340-x)
Supplement: Supplementary file 1 — Additional file 1: Fig. S1. Significantly longer OS among patients undergoing first-line allo-HSCT compared to first-line IST after excluding four studies with high heterogeneity. [file 12865_2020_340_MOESM1_ESM.docx]

**Allo-HSCT compared with immunosuppressive therapy for acquired aplastic anemia:** [**Is superiority a one-sided understanding?**](https://www.ncbi.nlm.nih.gov/pubmed/28884937)

Yangmin Zhu^1^, Qingyan Gao^1^, Jing Hu^1^, Xu Liu^1^, Dongrui Guan^1^, Fengkui Zhang^1^

^1^Department of Therapeutic Center of Anemia, Institute of Hematology and Blood Diseases Hospital, Chinese Academy of Medical Science & Peking Union Medical College (CAMS & PUMC), Tianjin, China


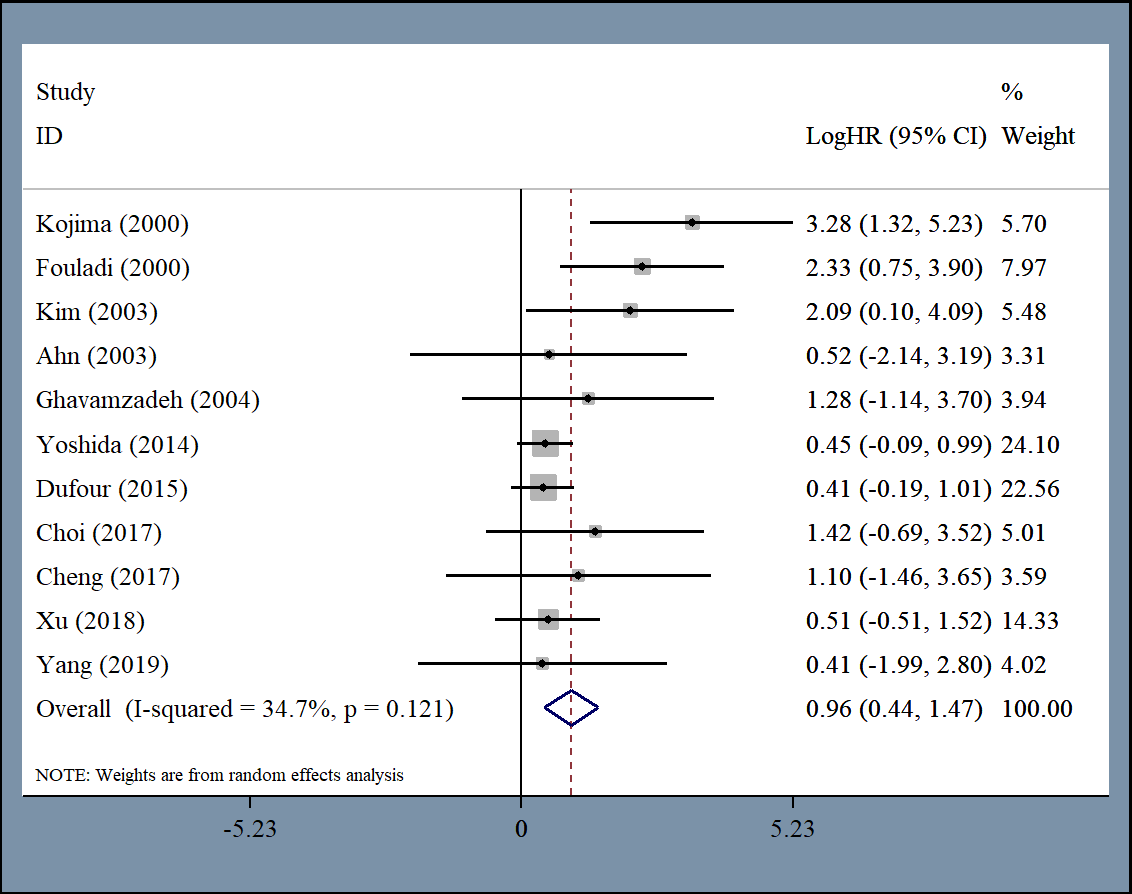


**Figure S1.** Significantly longer OS among patients undergoing first-line allo-HSCT compared to first-line IST after excluding four studies with high heterogeneity.
